# Trans. Ohio State Med. Society

**Published:** 1871-12

**Authors:** 


Transactions of the twenty-sixth annual meeting of the Ohio State Medical Society, held at Cincinnati, April 4th, 5th and 6th, 1871. Cincinnati: Bosworth, Case & Hall, publishers.

This is a handsome volume of 350 pages in substantial cloth binding. The table of contents is as follows: Minutes of Twenty-sixth Annual Meeting; Address of the Retiring President; Some Points in Uterine Therapeutics, by Edw. B. Stevens, M. D.; Report on Physical and Vital Force, by S. S. Scoville, M. D., Lebanon, Ohio; Report on the Antagonistic Power of Opium and Belladonna, by John A. Little, M. D., Delaware, Ohio; Case of Removal of Both Superior Maxillary Bones, by W. H. Mussey, M. D., Cincinnati; Sanitary Science, by J. R. Black, M. D., Newark, Ohio; Hydrate of Chloral, by D. D. Bramble, M. D., Cincinnati; Report on Opthalmology, by W. W. Seeley, M. D., Cincinnati; The Opthalmoscope and the Sphygmograph in the Study of the Physiological Action of Medicine, by Robert Bartholow, M. D., Cincinnati; Reproduction, by James T. Whittaker, M. D., Cincinnati; Prevailing Diseases, by J. R. Black, M. D., Newark, Ohio; Relations of Epilepsy, Insanity and Jurisprudence, by W. J. Conklin, M. D. Dayton, Ohio; Mechanical Treatment of Strictures of the Urethra, by D. S. Young, M. D., Cincinnati; Appendix to a "Historical Review," by Edw. B. Stevens, M. D., Cincinnati; Constitution of Ohio State Medical Society; Code of Ethics of American Medical Association.

The character of these reports and essays is such as to constitute a very valuable and important volume, highly creditable to the society by which it is issued.

